# [Expression of Concern] Curcumin suppresses breast tumor angiogenesis by abrogating osteopontin-induced VEGF expression

**DOI:** 10.3892/mmr.2026.13817

**Published:** 2026-02-04

**Authors:** Goutam Chakraborty, Shalini Jain, Smita Kale, Remya Raja, Santosh Kumar, Rosalin Mishra, Gopal C. Kundu

Mol Med Rep 1: 641–646, 2008; DOI: 10.3892/mmr_00000005

Following the publication of the above paper, it was drawn to the Editor's attention by a concerned reader that the statistical analysis in this study may not have employed the most appropriate statistical tests; namely, the paired Student's t-test was used for comparisons between independent groups, which the reader considered may have inflated the statistical significance. Neither may the paired Student's t-test have been the most appropriate test to have been selected for various of the migration and invasion assay experiments, wherein at least three groups were being compared.

Owing to the fact that the Editorial Office has been made aware of the possibility of inappropriate statistics handling in this paper, we are issuing an Expression of Concern to notify readers of this potential problem while the Editorial Office continues to investigate this matter further.

